# Balancing selection and genetic drift at major histocompatibility complex class II genes in isolated populations of golden snub-nosed monkey (*Rhinopithecus roxellana*)

**DOI:** 10.1186/1471-2148-12-207

**Published:** 2012-10-19

**Authors:** Mao-Fang Luo, Hui-Juan Pan, Zhi-Jin Liu, Ming Li

**Affiliations:** 1 Key laboratory of Animal Ecology and Conservation Biology, Institute of Zoology, Chinese Academy of Sciences, 1-5 Beixhenxi Road, Chaoyang, Beijing, 100101, China; 2 Key Laboratory of Vegetation and Environmental Change, Institute of Botany, Chinese Academy of Sciences, Beijing, 100093, China; 3 Graduate School of the Chinese Academy of Sciences, Beijing, 100049, China; 4 College of Nature Conservation, Beijing Forestry University, Beijing, 100083, China

**Keywords:** Balancing selection, Conservation genetics, Gene drift, MHC, *Rhinopithecus roxellana*

## Abstract

**Background:**

Small, isolated populations often experience loss of genetic variation due to random genetic drift. Unlike neutral or nearly neutral markers (such as mitochondrial genes or microsatellites), major histocompatibility complex (MHC) genes in these populations may retain high levels of polymorphism due to balancing selection. The relative roles of balancing selection and genetic drift in either small isolated or bottlenecked populations remain controversial. In this study, we examined the mechanisms maintaining polymorphisms of MHC genes in small isolated populations of the endangered golden snub-nosed monkey (*Rhinopithecus roxellana*) by comparing genetic variation found in MHC and microsatellite loci. There are few studies of this kind conducted on highly endangered primate species.

**Results:**

Two MHC genes were sequenced and sixteen microsatellite loci were genotyped from samples representing three isolated populations. We isolated nine *DQA1* alleles and sixteen *DQB1* alleles and validated expression of the alleles. Lowest genetic variation for both MHC and microsatellites was found in the Shennongjia (SNJ) population. Historical balancing selection was revealed at both the *DQA1* and *DQB1* loci, as revealed by excess non-synonymous substitutions at antigen binding sites (ABS) and maximum-likelihood-based random-site models. Patterns of microsatellite variation revealed population structure. *F*_ST_ outlier analysis showed that population differentiation at the two MHC loci was similar to the microsatellite loci.

**Conclusions:**

MHC genes and microsatellite loci showed the same allelic richness pattern with the lowest genetic variation occurring in SNJ, suggesting that genetic drift played a prominent role in these isolated populations. As MHC genes are subject to selective pressures, the maintenance of genetic variation is of particular interest in small, long-isolated populations. The results of this study may contribute to captive breeding and translocation programs for endangered species.

## Background

Understanding how levels of genetic variation influence the survival of threatened species is of fundamental interest to evolutionary and conservation biologists because many natural populations are threatened by intense reduction and fragmentation of habitat, leading to isolation, declining populations, and decreasing genetic diversity [1,2]. Loss of genetic diversity may increase the risk of extinction due to decreased reproductive fitness, decreased adaptive flexibility, and increased disease susceptibility [3]. Clarifying the mechanism that determines genetic variation in small, isolated populations is therefore essential for their conservation [4]. An important assumption in conservation genetics is that small, isolated populations are more sensitive to genetic drift and inbreeding [5,6]. Genetic drift is the random fluctuation of allele frequencies over time; thus, adaptive alleles may be lost and deleterious alleles could be fixed in the population. The small population size and fixation of deleterious alleles leads to inbreeding depression and reduction of individual fitness, which decreases viability and compromises a population’s evolutionary adaptive potential [6].

However, some functionally important genes that are maintained by balancing selection, such as major histocompatibility complex (MHC) genes, may have a different evolutionary pattern compared with neutral markers. The multi-gene MHC family is found in vertebrates, codes for cell surface glycoproteins, and is important in animal conservation due to its role in resisting pathogens [7]. Compared to nearly neutral markers such as microsatellite loci or mitochondrial DNA, which are informative for phylogenetic and phylogeographic reconstructions [8], MHC variability is believed to determine the capability of individuals to resist continuously evolving pathogens and parasites. Consequently, MHC variability is a reflection of the processes that are related to adaptive evolution within and between populations [9]. Thus, most variation at MHC loci reflects the effects of balancing selection [10], which is the main mechanism for retaining high MHC genetic diversity. Balancing selection includes frequency-dependent selection, overdominance and diversifying selection and promotes long evolutionary persistence of individual alleles and strongly differentiated allelic lineages in mammals [2]. Besides balancing selection, intragenic recombination has also been suggested as one evolutionary mechanism for generation of MHC sequence diversity [11,12]. Still, several significant questions remain. Current results conflict or are unclear with regard to the relative roles of balancing selection and genetic drift in maintaining MHC polymorphism in small, isolated, or severely bottlenecked populations [13,14]. Studies of guppies, Mexican wolves and Namibian leopards found that even when genetic diversity at neutral markers was poor, polymorphisms in MHC were still maintained by balancing selection [13-17]. In contrast, other research has found low levels of detectable polymorphisms for MHC genes in populations with lower diversity in neutral markers, including studies on fallow deer (*Cervus dama*) [18], northern elephant seals (*Mirounga angustirostris*) [19], great crested newt (*Triturus cristatus*) [20], and the black-footed rock-wallaby (*Petrogale lateralis lateralis*) [21]. These results suggest that compared to genetic drift balancing selection is relatively weak in small populations, leading to reduced variation at some MHC loci [22]. To elucidate the role of balancing selection and genetic drift in populations, *F*_ST_ outlier analysis [23] is widely used because demographic processes affect neutral loci and lead to population differentiation (measured by *F*_ST_) [24]. The *F*_ST_ values are computed for all genes to distinguish the genes under selection from those non-selective genes [25].

**Figure 1:**
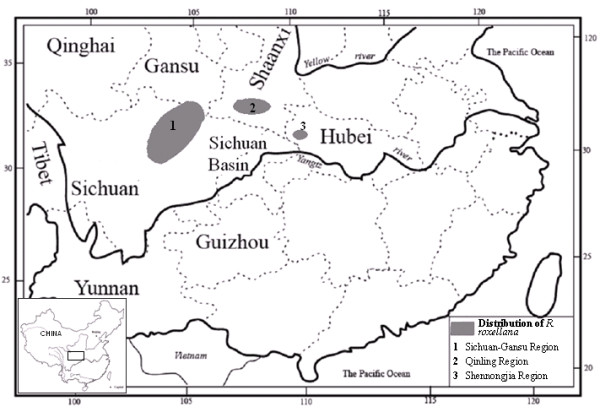
Distribution of isolated snub-nosed monkey populations.

We studied MHC and neutral genetic variation in the golden snub-nosed monkey (*Rhinopithecus roxellana*), an endangered primate endemic to China where it inhabits three isolated areas: Sichuan and Gansu provinces (SG); the Qinling Mountains, Shaanxi province (QL) and the Shennongjia Forestry District, Hubei province (SNJ) (Figure 1). Current census data suggest that fewer than 22,000 individuals remain (about 15,000 individuals in SG, 5500 individuals in QL and 1000 individuals in SNJ) [26], and mitochondrial DNA and microsatellite studies indicate that the genetically distinct SNJ population has very low genetic variation [27,28]. The current effective population size (*N*e) in SNJ is approximately 80 individuals [29]. The small size and isolation that typically characterize local populations make the golden snub-nosed monkey particularly susceptible to loss of genetic variation through inbreeding and genetic drift [30,31]. In this study, we 1) investigated population structure and polymorphism levels of 16 microsatellite loci and 2 MHC II genes, 2) tested for both selection at the MHC loci and patterns of between population differentiations, and 3) evaluated the role of balancing selection and genetic drift in populations.

## Results

### Genetic variation at microsatellites

Genotyping of the 16 microsatellite loci revealed that the highest level of expected heterozygosity (*H*_E_) and observed heterozygosity (*H*_O_) was in the SG population, while the lowest level was in the SNJ population (Table 1, Additional file 1: Table S1). The Hardy–Weinberg equilibrium test showed no deviation from equilibrium after Bonferroni correction. No linkage disequilibrium occurred for pairs of loci in any population, and Micro-Checker revealed no evidence of stuttering, null alleles, and allele dropout.

**Table 1:** Population genetic parameters for populations estimated from microsatellite data

**Population**	**N**	**No. of Loci**	**A**_**R**_	***H***_**E**_**±SD**	***H***_**O**_**±SD**	***F***_**IS**_
SG	25	16	4.635	0.736±0.022	0.714±0.022	0.017
QL	22	16	4.854	0.713±0.036	0.653±0.024	0.071
SNJ	17	16	3.473	0.611±0.038	0.591±0.030	0.030
All	64	16	4.321	0.755±0.022	0.660±0.014	0.041

*A*_R_, allelic richness; *H*_E_, expected heterozygosity; *H*_O_, observed heterozygosity; *F*_IS_, inbreeding coefficient.

### MHC variation, expression analyses, and recombination analysis

For each locus, we analysed 894 clones from 64 individuals that represented three monkey populations. We obtained 13 different *DQA1* fragments with lengths that ranged from 436 bp to 444 bp including exon 2 and partial intron 2 and intron 3 [GenBank: JQ217094-JQ217106]. After performing BLAST with the *HLA-DQA1* and *Macaca mulatta DQA1* exon 2, we removed the intron and obtained 9 *DQA1* exon 2 sequences with an equal length of 249 bp. These sequences were labelled as Rhro-DQA1*01–09 [GenBank: JQ217107-JQ217115] according to the nomenclature of Klein *et al.*[32] and were used for analysis. At the *DQB1* locus, we identified 16 unique *DQB1* exon 2 sequences that we called Rhro-DQB1*01–16 [GenBank: JQ217116- JQ217131].

Fragments of MHC loci were successfully amplified from the cDNA of 2 blood samples that were also used in DNA amplifications. At the *DQA1* locus, 189 bp fragments of *DQA1* exon 2 were obtained from 2 blood samples, and these fragments were part of the alleles obtained from DNA in the same individual [EMBL: HE616682- HE616683]. At the *DQB1* locus longer fragments were obtained, including the entire exon 2, partial exon 1, and exon 3; however, these fragments were only obtained from 1 blood sample [GenBank: JQ217132-JQ217133]. Shorter fragments of *DQB1* exon 2 were obtained from 2 blood samples [EMBL: HE616684- HE616686]. No sequences contained stop codons. The sequences from RNA were identical with that from DNA, which supports the hypothesis that these MHC PCR products were transcribed and expressed.

No more than two alleles were present per individual, suggesting that for each MHC locus one gene copy was sequenced with these primer sets. No stop codon or insertion/deletion was detected in *DQA1* and *DQB1*. The measures of MHC diversity in the 3 populations are summarized in Table 2. All three populations shared four of the nine *DQA1* alleles and two of the among 16 *DQB1* alleles (Additional file 2: Table S2). The MHC sequences were highly divergent: 61 of 249 bp of *DQA1* and 70 of 256 bp of *DQB1* were variable. The Hardy–Weinberg equilibrium tests showed that the QL population deviated from equilibrium (p = 0.006). Linkage disequilibrium occurred in the SG population (p = 0.01). Population recombination analysis using LDhat revealed that the *DQB1* locus had a higher recombination rate (ρ = 7) when all alleles were included between the MHC loci (all DQA alleles: ρ = 2).

**Table 2:** Summary of MHC variation in *R. roxellana *populations

**MHC**	**Population**	**N**	**Variable sites**	**Parsimony- informative sites**	**Total alleles**	**Private alleles**	**H**	**A**_**R**_	**Over mean distance**	***F***_**IS**_
*DQA1*	SG	25	59	29	6	1	0.798	5.628	0.118±0.016	0.675
	QL	22	48	30	8	3	0.851	7.661	0.090±0.013	0.511
	SNJ	17	43	7	4	0	0.671	4.000	0.090±0.015	0.123
	All	64	61	33	9	-	0.972	7.503	0.102±0.014	0.495
*DQB1*	SG	25	65	32	7	3	0.768	6.126	0.115±0.015	0.325
	QL	22	57	36	11	6	0.861	9.962	0.085±0.012	0.452
	SNJ	17	47	20	5	2	0.743	5.000	0.092±0.014	0.251
	All	64	70	56	16	-	0.891	9.587	0.097±0.012	0.360

Private alleles, alleles only found in this population; h, genetic variation; A_R_, allele richness; *F*_IS_, inbreeding coefficient.

### Population structure and phylogenetic analysis

**Figure 2:**
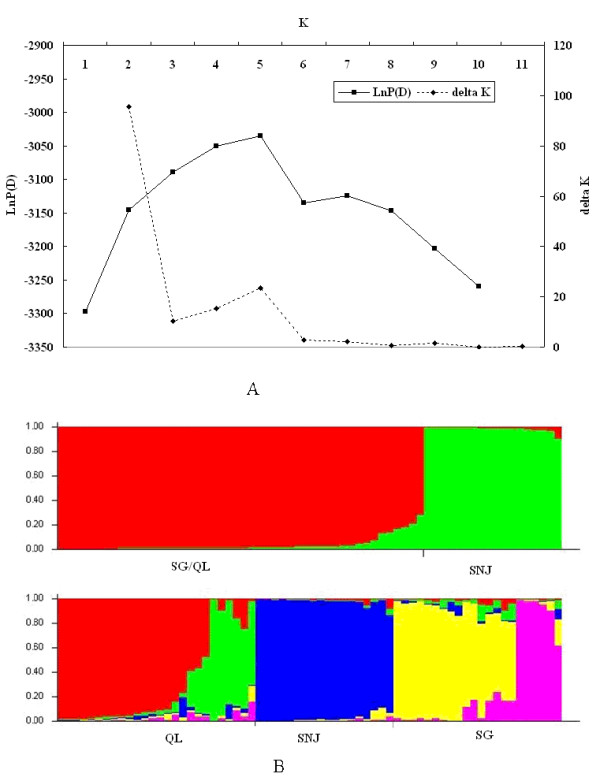
**Bayesian STRUCTURE clustering based on microsatellite genotypes among 3 snub-nosed monkey populations.** (**A**) Δ*K* values as a function of *K* based on 10 runs, indicating the most likely number of 2 genetic clusters. (**B**) STRUCTURE assignment output at *K* = 2 and *K* = 5. The proportions of ancestry assigned to different clusters were plotted by individuals.

**Figure 3:**
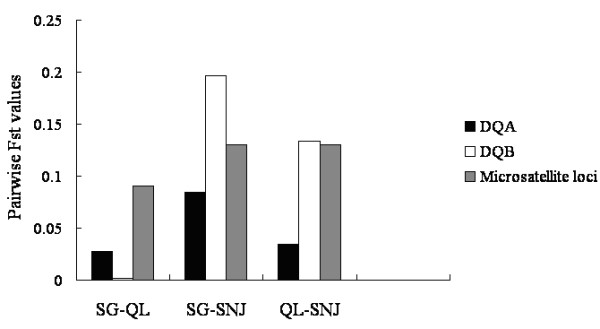
Differences of pairwise *F*_**ST **_values among populations in MHC genes and microsatellite loci.

**Figure 4:**
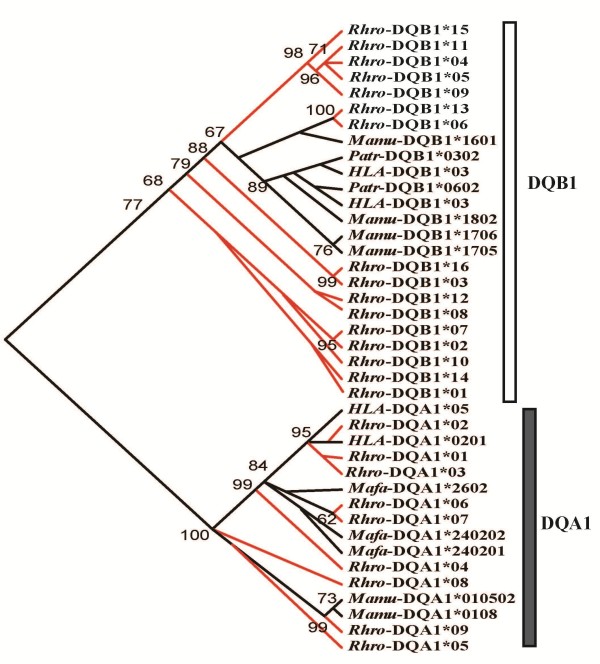
**Phylogenetic relationships among snub-nosed monkey MHC alleles.** The phylogenetic tree was reconstructed using the ML inference method. Bootstrap values above 60 are given for each clade. The alleles obtained in this study are shown as red bars. Other allelic sequences, which were downloaded from GenBank are included in the analyses: *Homo sapiens* (HSU97555, AY375917, GQ422610, AY334565); *Macaca fascicularis* (AM086058-AM086060); *Macaca mulatta* (M81297, M81292, AJ308047, AJ308046); *Pan troglodytes* (M81260, M81262).

Microsatellite-based analyses detected significant genetic structures among the populations (Figure 2). Two genetic clusters were found (SG-QL, SNJ) with a clear maximum Δ*K* (Δ*K* = 95.500 at *K* = 2). The pairwise *F*_ST_ was the largest between SNJ and the other 2 populations among all of the *F*_ST_ values (Figure 3), which showed that the differences between SG and QL were minimal compared to those between SG-QL and SNJ. However, there was also a small Δ*K* peak at *K* = 5 (Δ*K* = 23.59), and results showed that in addition to the divergence between SG and QL, there was also divergence within the SG and QL populations. These results agree with earlier studies, where the differences within the SG and QL populations resulted from their different origins and habitat fragmentation [33,34]. The clear population structure observed in microsatellites was not found at either the *DQA1* locus or the *DQB1* locus (Additional file 3: Figure S1). Similar to previous studies [7], the relationship among MHC alleles did not agree with the geographical locations of the 3 populations, as observed in the microsatellite results (Figure 4). *DQA1/DQB1* alleles were not grouped by species but were mixed with other species (trans-species polymorphism (TSP). No clear clade could be identified from the phylogenetic tree at these two loci. Populations had more private alleles at *DQB1* than *DQA1* (Table 2). Four among 9 *DQA1* exon 2 alleles were commonly shared among the 3 populations, while just 2 (Rhro-DQB1*01, 03) among 16 *DQB1* exon 2 alleles were shared among all of the populations (Additional file 2: Table S2). However, frequency differences of these shared alleles were found between all population pairs (except Rhro-DQA1*01, 02). Comparisons of pairwise *F*_ST_ values of MHC and microsatellite sites are shown in Figure 3. The *F*_ST_ values showed lower divergence among populations compared with microsatellite loci (except SG-SNJ and QL-SNJ at the *DQB1* locus). Half of the *F*_ST_ values at the MHC loci were higher than 0.05, indicating that differences existed among populations, especially between SG and SNJ. The population difference was more distinct at the *DQB1* locus: the differentiations between the SG and SNJ populations and between the QL and SNJ populations were even larger than the differentiation at the microsatellite.

### Historical selection

Evidence for historical balancing selection was detected. First, in the ABS, the number of nonsynonymous (*d*_N_) mutations was greater than the number of synonymous (*d*_S_) mutations using the Z-test (*DQA1*: *d*_N_/*d*_S_ = 1.15, p = 0.405; *DQB1*: *d*_N_/*d*_S_ = 4.41, p = 0.01) (Table 3). Although p-value was not significant for *DQA1* locus in Mega, ABS sites under significant selection were detected in Paml 4 (Additional file 4: Table S3). After comparisons of various codon evolution models using Codeml in Paml 4, models integrating positive selection (M2a, M8, and M3) matched MHC better than the other models based on the Akaike information criterion (AIC) values (Tables 4 and 5). Under model M2a, 9 *DQA1* sites and 5 *DQB1* sites were exposed to significant selection. Under model M8, 11 *DQA1* sites and 9 *DQB1* sites were identified. Moreover, most of these sites were ABS sites and others located near ABS sites (Additional file 4: Table S3, Additional file 5: Table S4). The existing TSP at these loci provided additional evidence in support of historical balancing selection (Figure 4).

**Table 3:** Average non-synonymous substitutions per non-synonymous site (*d*_**N**_) and synonymous substitutions per synonymous sites (*d*_**S**_)

**MHC**	**Sites**	**N**	***d***_**N**_	***d***_**S**_	***d***_**N**_**/*d***_**S**_	**Z**	**P**
*DQA1*	ABS	20	0.152±0.048	0.132±0.080	1.15	0.247	0.405
	Non-ABS	62	0.081±0.020	0.113±0.031	0.72	−0.882	0.380
	All	82	0.100±0.020	0.114±0.026	0.88	−0.426	0.671
*DQB1*	ABS	23	0.247±0.066	0.056±0.033	4.41	2.477	0.01
	Non-ABS	62	0.063±0.014	0.092±0.022	0.68	−1.104	0.272
	All	85	0.103±0.018	0.083±0.017	1.24	0.709	0.480

**Table 4:** Results of maximum-likelihood models for exon 2 of the *DQA1 *gene

**Model code**	**P**	**Log-likelihood**	**Parameter estimates**	**Positively selected sites**
M0(one ratio)	1	−774.550	ω=0.643	None
M1a(nearly neutral)	1	−760.293	p_0_= 0.723 (p_1_= 0.277)	Not allowed
M2a(positive selection)	3	−752.443	p_0_= 0.911, p_1_= 0.033(p_2_= 0.056) ω_2_= 5.148	*13T*,**16F,**35G,42R,*45E*,46L,48K,**50G,54P,**56G,59R,**61L,63T,64S,70I,**71M,74R
M3(discrete)	4	−752.206	p_0_= 0.736, p1= 0.244 (p_2_= 0.018) ω_1_= 1.298, ω_2_= 8.174	Not allowed
M7(beta)	2	−761.268	p= 0.008, q= 0.028	Not allowed
M8(beta and omega)	4	−752.248	p_0_= 0.974 (p_1_= 0.025) p= 0.008, q= 0.023, ω= 7.292	10L,**13T,16F,29Q,35G,42R,43W,45E,**46L,48K,**50G,**51G,54P,56G,59R,**61L,***63T*,*64S*,70I,71M,74R,79A

P is the number of parameters in the ω distribution, ω is the selection parameter, and p_n_ is the proportion of sites falling into the ω_n_ site class. For models M7 and M8, p and q are the shape parameters of the β function. Positively selected sites were identified in models M2a and M8 by the Bayes empirical Bayes procedure (Yang *et al.* 2005). Sites inferred under selection at the 99% level are listed in bold, and those inferred at the 95% level are shown in italics.

**Table 5:** Results of maximum-likelihood models for exon 2 of the *DQB1 *gene

**Model code**	**P**	**Log-likelihood**	**Parameter estimates**	**Positively selected sites**
M0(one ratio)	1	−1067.274	ω=0.496	None
M1a(nearly neutral)	1	−1007.026	p_0_= 0.869 (p_1_= 0.131)	Not allowed
M2a(positive selection)	3	−989.138	p_0_=0.818, p_1_=0.176(p_2_= 0.006) ω_2_= 8.276	**1V**,6M,**18L**,20T,48S,*49S*,67L,*72R*,**77L**,79L,81T
M3(discrete)	4	−994.628	p_1_= 0.059 (p_2_= 0.018) ω_1_= 0.076, ω_2_= 3.390	Not allowed
M7(beta)	2	−1007.600	p= 0.006, q= 0.036	Not allowed
M8(beta and omega)	4	−989.309	p_0_= 0.994 (p_1_= 0.006) p= 0.005, q= 0.020, ω= 8.643	**1V**,6M,**18L**,*20T*,22Y,29Y,30A,38E,*48S*,**49S**,62G,63T,67L,*72R*,**77L**,*79L*, *81T*

P is the number of parameters in the ω distribution, ω is the selection parameter, and p_n_ is the proportion of sites falling into the ω_n_ site class. For models M7 and M8, p and q are the shape parameters of the β function. Positively selected sites were identified in models M2a and M8 by the Bayes empirical Bayes procedure (Yang *et al.* 2005). Sites inferred under selection at the 99% level are listed in bold, and those inferred at the 95% level are shown in italics.

### *F*_ST_ outlier analysis

**Figure 5:**
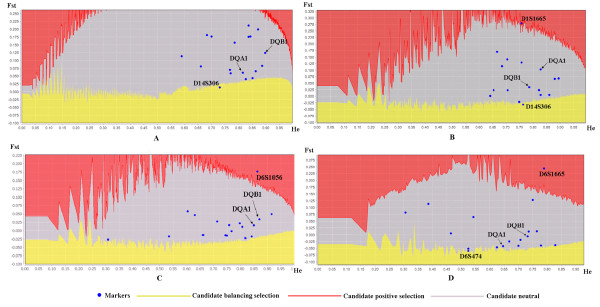
*F*_**ST **_outlier analysis. Circles are observed values from 16 microsatellite loci and MHC genes for (A) all 3 populations, (B) SG population, (C) QL population, and (D) SNJ population.

The *F*_ST_ outlier analysis did not reveal a high or low level of differentiation among populations at the *DQA1* and *DQB1* loci compared with 16 microsatellite loci (Figure 5A). One microsatellite locus (D14S306) was located outside the 95% confidence interval. For each population, the MHC loci were also located within the 95% confidence interval of the microsatellites. Some satellites existed outside the 95% confidence interval: in the SG population, D14S306 was lower than the neutral level, and D1S1665 was at the edge of the neutral level (Figure 5B); in the QL population, D6S1056 was under the candidate positive selection level (Figure 5C); and in the SNJ population, D1S1665 was above the neutral level while D6S474 was below (Figure 5D).

## Discussion

Habitat isolation generates barriers to gene flow among populations that often result in loss of genetic diversity through genetic drift and inbreeding [2]. For vertebrates, genetic variation is of special importance in MHC genes due to the significant role they play in immune functions [35]. MHC diversity is presumed to improve parasite resistance, reproductive success, and population viability [36], and has been studied in species such as western gorilla (*Gorilla gorilla*) [37], brown bear (*Ursus arctos*) [38], Ethiopian wolf (*Canis simensis*) [39], European bison (*Bison bonasus*) [40], Bengal tiger (*Panthera tigris tigris*) [41], and Namibian leopard (*Panthera pardus pardus*) [17]. In this study, a relatively high level of MHC variation was found in the golden snub-nosed monkey, with 9 *DQA1* and 16 *DQB1* alleles found in 64 individuals. However, we found lower MHC variation in the SNJ population, which also showed lower genetic diversity in microsatellites and mitochondrial genes [27,34]. Small sample size cannot account for the reduced variability, because sample collected across the entire distribution of the SNJ population showed similar patterns of genetic diversity [29].

For each MHC locus, regardless of how many clones were sequenced from an individual, no more than 2 alleles were observed in an individual, a strong indicator that we amplified single loci in all cases. We assumed that all alleles were from a single functional gene. The assumption that our sequences were from functional genes was supported by three findings: 1) sites inferred to have been exposed to significant selection, most of which were ABS sites, indicating historical selection at functional genes; 2) no reading frame or stop codon disruptions found in any alleles; and 3) the sequences identified from cDNA were parts of or included sequences obtained from DNA.

### Historical balancing selection

Golden snub-nosed monkey MHC genes reflect historical balancing selection in that an excess of non-synonymous substitutions was mainly concentrated in the ABS (Table 3, Additional file 4: Table S3, Additional file 5: Table S4). According to neutrality theory [42], the synonymous nucleotide substitution rate is larger than the non-synonymous substitution rate because a change in amino acid sequence has a greater possibility of being deleterious. The elevated rate of non-synonymous substitutions at the ABS provided clear evidence of positive selection [43,44] shaping genetic variation [45]. The p-value was not significant at *DQA1* ABS may due to a weaker recombination within *DQA1* as a lower recombination rate has been shown before. Without higher recombination and stronger selection, some *DQA1* alleles might be lost when population size decreased. The sharing of MHC alleles among populations also indicates that MHC alleles may have been conserved by selection [19]. Second, random site models analysis proved the existence of historical selection based on the maximum likelihood method, which revealed that, for MHC genes, the models including selection (M2a, M3, and M8) match MHC alleles better than models without selection (Table 4,and 5). Under the M2a and M8 models, some sites of the 2 MHC loci were under significant selection pressure. Furthermore, trans-species evolution of the MHC alleles revealed historical balancing selection. Under balancing selection, some MHC alleles or allelic lineages are reported in other species, which indicates that they are ancestral alleles [46].

Genes vary in terms of the level of selection, and both *DQA1* and *DQB1* revealed different patterns of selection. Each population had unique *DQB1* alleles, while not every population had unique *DQA1* alleles (Table 2). Population divergence, measured as pairwise *F*_ST_, was larger in *DQB1* than in *DQA1* except for that between SG and QL (Figure 3). Similar results were reported in water vole, where balancing selection pressure was different at MHC genes in continuous populations [47]. MHC genes are assumed to be closely linked [48]. In our study, however, linkage disequilibrium between *DQA1* and *DQB1* was only observed in the SG population. This weak linkage disequilibrium and the different selection pressures on these loci could be a result of recombination, which is common at the MHC genes [49,50]. The higher recombination rate that was found in *DQB1* genes may explain their larger allelic richness compared to *DQA1* genes. Recombinants maintained by selection may counteract the linkage of closely linked genes [51] and play an important adaptive role in *DQB1* evolution. In the present study, historical selection was found, but this does not conclusively indicate that balancing selection is acting on current populations. First, an excess of non-synonymous mutations requires a long time to accumulate [47]. Once present, this pattern would take a long time to vanish after the disappearance of selection [10]. Hence, we investigated whether selection continues to play a major role at present.

### Patterns of selection and drift

Although selection historically maintained MHC diversity, recent population isolation and fragmentation has increased the role of genetic drift in shaping patterns of MHC variation in snub-nosed monkeys. First, compared with neutral forces, balancing selection is supposed to diminish population differentiation as measured by conventional pairwise *F*_ST_[22,52]. Thus, the population structure of genes under balancing selection should not be pronounced [53]. However, in the present study, half of the pairwise *F*_ST_ values were greater than 0.05, and two *F*_ST_ values at DQB were even greater than those at microsatellites (Figure 3). Second, *F*_ST_ outlier analysis showed that the structure in the MHC loci was within the neutrality level for all populations and for each population. Considering all populations, one microsatellite (D14S306) showed a *F*_ST_ value that was lower than the neutral level, indicating its linkage with other genes under selection [22]. Lastly, a positive correlation was found between allelic richness in MHC and microsatellites. The QL population had the highest allelic richness in microsatellites and MHC, while SNJ had the lowest. This positive correlation indicates that genetic drift plays a significant role in maintaining MHC diversity for snub-nosed monkeys [25]. Maintenance of MHC variation through balancing selection may be hampered in small, isolated populations because of their lower effective recombination rate [54]. In all, our results indicate that even though selection acts on MHC, it is overwhelmed by genetic drift in small, isolated populations.

The positive correlation in the allelic richness of MHC and microsatellites, together with other evidence, indicates that genetic drift has a great influence on the maintenance of MHC variations in small, isolated populations of snub-nosed monkeys [7,25,55]. No evidence showed that MHC polymorphism had increased in populations that contained low neutral variation [27,42]. Under neutral evolution theory [56], alleles are expected to be neutral when s < 1/2 *N*e (s = selection coefficient, *N*e = effective population size). Therefore, the smaller *N*e becomes, the greater the likelihood of genetic drift [57]. The SNJ population is subject to more genetic drift than the other two populations as found in a previous study [29]. Other animals whose patterns of MHC polymorphism have been contributed to drift over selection include the great crested newt (*Triturus cristatus*) [19], black-footed rock-wallaby (*Petrogale lateralis lateralis*) [20], tuatara (*Sphenodon spp.*) [7], and the Egyptian vulture (*Neophron percnopterus*) [58]. These results suggest that selection on MHC is not strong enough to counteract drift that results from population fragmentation, isolation and bottleneck.

## Conclusion

Variation at MHC loci is widely accepted as being maintained by balancing selection (reviewed in 2), even with a low level of neutral variability in some species [29,59]. This shows the importance of balancing selection for maintaining variation in the field and exposes the problem of using neutral genes as substitutes for variation in fitness-related genes [32]. However, in small, isolated populations or bottlenecked populations, balancing selection is overwhelmed by drift [21]. In this study, we found the same genetic variation pattern both at neutral and MHC markers, suggesting that genetic drift was stronger than selection, thus leading to a reduction in MHC diversity in the most isolated populations. Such findings may contribute to the conservation of endangered species such as snub-nosed monkeys both in captive breeding and translocation programs. Though the relationships between MHC with mate choice and pregnancy outcomes still remain controversial [60], it could be helpful to examine MHC variation in captive individuals that are involved in mating programs. In the field, translocation of individuals from demographically and genetically healthy populations to populations that suffer from reduced genetic diversity can improve the chances of genetic recovery [61,62]. Recently, researchers have found that inbred populations could thrive with the import of migrants as part of a conservation management program [63-65]. Further, the introduction of individuals from western populations of golden snub-nosed monkeys could restore genetic diversity to the relatively homogenous SNJ groups, reducing the likelihood of inbreeding depression.

## Methods

### Sample collection and DNA extraction

We collected 64 *R. roxellana* samples (muscle, skin, and blood) from the current population (SG population = 25, SNJ population = 17, and QL population = 22) (Figure 1 and Table 1). All collections complied with the relevant animal welfare institutions and laws of China. Also, the Institute of Zoology (Chinese Academy of Sciences) provided ethical approval for this study. Muscle and skin samples were collected from carcasses that were provided by local museums and nature reserves. Skin samples were stored dry, muscle samples were stored in 95% ethanol, and blood samples were collected while trapping individuals for physical examination and were stored in a refrigerator at −80°C. During DNA extraction, benches and plasticware were washed with 10% bleach and sterile water and were exposed to UV light for 30 min prior to treatment to prevent contamination during DNA extraction. During the subsequent polymerase chain reaction (PCR), 8 extraction controls were used without any positive amplification.

### Microsatellite analyses

Samples were genotyped at 16 microsatellite loci [33]. Thirty-five cycles of PCR amplification were executed at the same time for up to 3 loci with reliable genotypes, with combinations selected by fluorescent dye (HEX, ISMRA, or FAM), Tm, and fragment size using the QIAGEN Mutliplex PCR kit following the manufacturer’s protocols at optimized annealing temperatures (55°C). An ABI 377 prism automated sequencer was used to resolve products, which were then analysed using GeneScan v3.1.2 and Genotyper 2.5 (Applied Biosystems).

### MHC amplification, cloning, sequencing, and expression analyses

The PCR was carried out in a 50 μL solution including 10 mM Tris–HCl (pH 8.4), 50 mM KCl, 2.5 mM MgCl_2_, 0.4 μM each primer, 0.2 mM each dNTP, 1.0 unit Hotstart-Taq DNA polymerase (Takara), and 10–100 ng DNA template. The amplification profile consisted of 5 min at 94°C, followed by 35 cycles of 30 s at 94°C, 30 s at 56°C (*DQA1*) or 58°C (*DQB1*), and 30 s at 72°C, with a final extension of 10 min at 72°C. The following primers were used for amplifying exon 2: 5'*DQA1*-AAGCCCA TAATATT TGAAAGTCAGT and 3'*DQA1*-TATGTGATTTTAGAGATGGGAGATG, or 5'*DQB1*-TGTAAAC GACGGCCAGTTCCCCGCAGAGGATTTCGTG and 3'*DQB1*-TGCTCTAGAGGGCGACGACGACGCCTCACCTC [66]. The *DQA1* primers were designed based on BLAST sequences from relative species. According to the manufacturer’s protocols, a Wizard PCR Preps DNA Purification Kit (Promega) was used to purify the PCR products. Purified PCR products were cloned using the pMD-18T vector (Takara) following manufacturer’s instructions. Ten to twenty clones containing inserts from each individual were sequenced on an ABI 377 or ABI-PRISMTM 3100 Genetic Analyzer (Applied Biosystems Inc.) with the Prism BigDyeTM Terminator Ready Reaction Kit (Applied Biosystems Inc.).

Expression analyses were conducted to validate the expression of the obtained MHC II-DQA and DQB alleles. The RNA was isolated from 2.5 mL whole blood using Trizol (Invitrogen) following the manufacturer’s instructions. To ensure that the genomic DNA was removed from the isolated RNA, a second DNA digestion was performed using the DNase I RNase-free Set (Promega). The cDNA synthesis was obtained using 200 U of M-MuLV Reverse Transcriptase (Promega) in a 25 μL reaction tube containing 1μL Oligo(dT)12–18 primer (0.5 μg/μL; Invitrogen), 2 μg total RNA as a template, and 25 U ribonuclease inhibitor, RNase-free water, 5 μL dNTP mix (10 mM), and 5 μL 5× reaction buffer. The reactions were incubated at 42°C for 60 min. The cDNA was acquired from 2 samples and was amplified by PCR using the primer sets (Additional file 6: Table S5). The primer sets were exon-spanning to detect the amplification of genomic DNA contaminants based on the BLAST results of human and *Macaca mulatta* exons. All amplified cDNA products were analysed by cloning and sequencing as described above.

### Data analysis

#### Microsatellite loci diversity analyses

Genetic diversity was measured as observed (*H*_O_) and expected heterozygosities (*H*_E_) [67]. The above analyses were computed using Arlequin v3 [68]. Inbreeding coefficients (*F*_IS_) and allelic richness (A_R_) analyses were performed in Fstat version 2.9.3 [69]. The A_R_ calculations were set to the smallest population size correction. Pairwise *F*_ST_ among populations and Hardy–Weinberg equilibrium were analysed across all loci for each population in an exact probability test in Genepop 4.0 [70]. Using the Bonferroni correction, significance values were adjusted for multiple comparisons. Linkage disequilibrium (LD) was calculated across all loci with the Weir correlation coefficient [71] in Genepop 4.0 [70]. To determine which LD values were significant, a permutation test was used. The presence of stuttering, null alleles, and allele dropout were examined using Micro-Checker [72].

#### MHC diversity analyses

Sequences were aligned and translated into the amino acid sequences in Mega 4 [73]. The gene identity was verified through homology with publicized MHC alleles of other species using BLASTN (http://blast.ncbi.nlm.nih.gov/Blast.cgi) from NCBI. Because some obtained sequences are false alleles potentially corresponded to PCR amplification artifacts [47], a new sequence was assumed to be an allele when it was identified from 3 separate amplications from the same sample or from at least 3 different samples [74]. We used Mega 4 to detect the variable and parsimony-informative sites (sites with at least 2 different nucleotides or amino acids), to compute the mean number of nucleotide differences, and to derive the overall mean genetic distances of nucleotide sequences. Standard estimate errors were detected using 1,000 bootstrap replicates. Hardy–Weinberg equilibrium tests, pairwise *F*_ST_ values, and LD were calculated in Genepop. The A_R_ and *F*_IS_ of *DQA1* and *DQB1* were compared among populations using the comparison among groups of samples option in Fstat version 2.9.3 [69]. The *A*_R_ estimates were adjusted to the smallest sample size.

#### Phylogenetic analysis and recombination analysis

To detect the genetic structures of microsatellite loci, Bayesian clustering was used in Structure 2.1 [75]. Because of the subtle population structure, correlated allele frequencies between populations and the admixture model were chosen [76]. The clusters (*K*) tested range was set from 1 to 10, and 10 independent runs were executed for each analysis. The Markov Chain Monte Carlo (MCMC) iterations lengths and burn-ins were set at 5,000,000 and 200,000, respectively. For most situations, Δ*K* had a mode at true *K*; thus, true K was selected using the Δ*K* statistic, which was calculated according to the rate of the log probability change of the data between successive *K* values [77].

The relationship of the MHC allele phylogenesis was constructed using a maximum likelihood method in PhyML 3.0 [78]. Before phylogenetic analysis, the most appropriate evolution models of sequence were estimated based on Akaike Information Criterion (AIC) in Modeltest 3.7 [79]. The K80 + G model was suggested to be the optimum model for MHC sequences with the gamma shape parameter α = 0.6532. The reliability of the obtained tree topology structure was carried out with 1,000 bootstrap replications. In addition, intraspecific phylogenetic structures were inferred using median-joining networks in Network 4.600 [80]. To estimate the rate of population recombination ρ (ρ = 4N_e_r), the composite-likelihood method [81] in LDhat [82] was used. The ρ is calculated using the crossing over for an effective population size (N_e_) and rate per generation (r), and is estimated without prior information [82]. Even for the sequences that evolved under balancing selection in the existence of recombination events, the LDhat still works efficiently [83].

### Detecting historical selection

Two methods were used to detect historical selection. First, we calculated the rates of non-synonymous and synonymous substitutions at all amino acid sites, ABS sites, and non-ABS in Mega 4 by the Nei-Gojobori method with the Jukes-Cantor correction [84] and 1,000 bootstrap replicates to obtain standard errors. The putative ABS and non-ABS locations were derived according to the structure of human MHC II [85]. Compare with the method that used HLA-DR1 [86] structure to derive ABS sites, Reche and Reinherz’s research provided a better way to avoid the emergence of uncertain gaps while blasting DQ1 genes with DR1 genes, and was also adopted by other studies [87,88]. Historical selection evidence was obtained with Codeml in the Paml 4 package [89]. This procedure examined heterogeneity in ω (ω = *d*_N_/*d*_S_) [90] among codons based on the maximum likelihood method, with positive selection indicated by ω = *d*_N_/*d*_S_ > 1. Six models (M0, M2a, M3, M7, M8) allowing for different selection intensity among sites were tested [91,92]. The sites revealed to be under selection were compared with ABS sites.

### Detecting recent selection

The *F*_ST_ outlier analysis [22] was used to examine whether MHC genes had significantly different population differentiation measures compared with microsatellite loci. Loci outside the confidence intervals of neutral *F*_ST_ values were likely under selection. The *F*_ST_ was estimated for *DQA1*, *DQB1*, and 16 microsatellite loci using Lositan [22,93]. Simulations involving 100 demes were sampled, and *H*_E_ and *F*_ST_ were printed for each of the 100,000 simulations. The infinite allele model was used in simulations that were performed using Fdist2. The aforementioned *F*_ST_ outlier analysis was also carried out on each population respectively. For each population, simulation parameters were similar using the same parameter set.

## Authors’ contributions

Luo MF carried out the molecular genetic studies, performed the statistical analysis, and drafted the manuscript. Pan HJ participated in the sequence alignment. Liu ZJ participated in the design of the study. Li M conceived the study, participated in its design and coordination, and helped to draft the manuscript. All authors read and approved the final manuscript.

## Supplementary Material

Additional file 1 **Table S1.** Microsatellite data of 64 samples. Click here for file

Additional file 2 **Table S2.** MHC haplotype distributions in populations. Click here for file

Additional file 3 **Figure S1.** Median-joining networks for MHC alleles of the snub-nosed monkeys. A) Network for *DQA1* alleles; B) Network for *DQB1* alleles. The circles represent alleles (SG population-red, QL population-yellow, SNJ population-blue), with the area proportional to the frequency of the alleles in the 3 populations. Click here for file

Additional file 4 **Table S3.** Alignment of the deduced amino acid sequences of *Rhro*-DQA1 exon 2 sequences. Identical amino acids are shown by points, * represent ABS site, and sites revealed to be under significant selection in PAML are shown by dash. Click here for file

Additional file 5 **Table S4.** Alignment of the deduced amino acid sequences of *Rhro*-DQB1 exon 2 sequences. Identical amino acids are shown by points, * represent ABS site, and sites revealed to be under significant selection in PAML are shown by dash. Click here for file

Additional file 6 **Table S5.** cDNA primer sets for *DQA* and *DQB* (primer sets with * were those that amplified successfully in cDNA). Their location within the gene is shown in Additional file 7: Figure S2. Click here for file

Additional file 7 **Figure S2.** Schematic representation of the position of cDNA primer sets used in the study of MHC II variation. Click here for file
