# Supplementary material for: Balancing selection and genetic drift at major histocompatibility complex class II genes in isolated populations of golden snub-nosed monkey (Rhinopithecus roxellana)
Source: BMC Evol Biol. 2012 Oct 19;12:207. doi: 10.1186/1471-2148-12-207 (PMC3532231; doi:10.1186/1471-2148-12-207)
Supplement: Additional file 1 — Table S1. Microsatellite data of 64 samples. [file 1471-2148-12-207-S1.doc]

**Table S1**

| ID | 1826 |  | 1665 |  | 442 |  | 817 |  | 1656 |  | 2204 |  | 474 |  | 611 |  | 1056 |  | 1432 |  | 306 |  | 493 |  | 1457 |  | 533 |  | 676 |  | 321 |  |
| --- | --- | --- | --- | --- | --- | --- | --- | --- | --- | --- | --- | --- | --- | --- | --- | --- | --- | --- | --- | --- | --- | --- | --- | --- | --- | --- | --- | --- | --- | --- | --- | --- |
| 1 | 149 | 149 | 192 | 196 | 323 | 339 | 137 | 137 | 147 | 147 | 252 | 252 | 150 | 158 | 287 | 291 | 286 | 290 | 144 | 152 | 184 | 192 | 270 | 270 | 127 | 127 | 209 | 213 | 191 | 195 | 190 | 214 |
| 2 | 145 | 149 | 188 | 192 | 315 | 335 | 137 | 141 | 131 | 135 | 248 | 260 | 150 | 158 | 279 | 279 | 274 | 298 | 144 | 144 | 180 | 192 | 266 | 274 | 131 | 135 | 209 | 209 | 191 | 203 | 190 | 190 |
| 3 | 149 | 149 | 188 | 196 | 315 | 331 | 137 | 137 | 143 | 147 | 252 | 260 | 150 | 158 | 287 | 291 | 290 | 294 | 148 | 152 | 188 | 192 | 266 | 270 | 127 | 127 | 213 | 213 | 191 | 195 | 214 | 218 |
| 4 | 145 | 149 | 188 | 200 | 319 | 319 | 141 | 141 | 131 | 147 | 260 | 284 | 154 | 170 | 287 | 295 | 270 | 282 | 148 | 156 | 184 | 192 | 266 | 274 | 123 | 127 | 213 | 213 | 195 | 199 | 206 | 210 |
| 5 | 145 | 149 | 188 | 196 | 311 | 311 | 133 | 137 | 131 | 147 | 252 | 272 | 154 | 154 | 283 | 299 | 270 | 274 | 144 | 152 | 184 | 188 | 266 | 270 | 123 | 127 | 209 | 213 | 191 | 191 | 210 | 210 |
| 6 | 141 | 149 | 188 | 200 | 327 | 331 | 137 | 141 | 147 | 147 | 248 | 248 | 150 | 158 | 283 | 283 | 270 | 282 | 144 | 144 | 184 | 192 | 266 | 266 | 123 | 135 | 213 | 213 | 191 | 195 | 214 | 218 |
| 7 | 149 | 149 | 188 | 188 | 319 | 327 | 137 | 137 | 147 | 147 | 256 | 260 | 150 | 154 | 287 | 299 | 274 | 298 | 156 | 156 | 180 | 184 | 266 | 274 | 127 | 131 | 213 | 217 | 199 | 199 | 190 | 214 |
| 8 | 145 | 149 | 188 | 188 | 327 | 331 | 137 | 141 | 143 | 147 | 248 | 264 | 134 | 134 | 279 | 303 | 270 | 274 | 152 | 156 | 180 | 180 | 266 | 274 | 127 | 131 | 213 | 221 | 195 | 195 | 190 | 218 |
| 9 | 145 | 145 | 188 | 188 | 331 | 335 | 129 | 133 | 147 | 147 | 248 | 256 | 158 | 158 | 279 | 279 | 270 | 298 | 144 | 152 | 184 | 188 | 266 | 270 | 123 | 127 | 213 | 221 | 199 | 203 | 190 | 210 |
| 10 | 149 | 149 | 188 | 196 | 319 | 327 | 133 | 137 | 131 | 147 | 248 | 256 | 154 | 154 | 287 | 299 | 282 | 298 | 152 | 156 | 184 | 188 | 270 | 274 | 127 | 131 | 213 | 221 | 195 | 199 | 214 | 218 |
| 11 | 145 | 149 | 188 | 196 | 319 | 327 | 133 | 137 | 131 | 147 | 248 | 260 | 150 | 154 | 283 | 287 | 270 | 282 | 144 | 156 | 188 | 192 | 270 | 270 | 123 | 123 | 213 | 213 | 195 | 199 | 214 | 214 |
| 12 | 145 | 149 | 188 | 188 | 319 | 319 | 137 | 141 | 147 | 147 | 264 | 276 | 150 | 158 | 279 | 287 | 278 | 282 | 144 | 148 | 188 | 192 | 270 | 274 | 135 | 135 | 213 | 217 | 195 | 203 | 190 | 214 |
| 13 | 149 | 149 | 188 | 200 | 331 | 335 | 137 | 141 | 131 | 147 | 248 | 260 | 142 | 142 | 279 | 295 | 270 | 282 | 148 | 156 | 184 | 196 | 270 | 274 | 123 | 127 | 213 | 213 | 195 | 195 | 190 | 210 |
| 14 | 145 | 145 | 196 | 200 | 223 | 327 | 133 | 137 | 127 | 131 | 248 | 296 | 158 | 170 | 295 | 299 | 294 | 298 | 148 | 156 | 184 | 188 | 266 | 270 | 123 | 131 | 213 | 213 | 191 | 199 | 210 | 218 |
| 15 | 145 | 145 | 196 | 196 | 327 | 327 | 133 | 133 | 131 | 143 | 252 | 252 | 154 | 162 | 295 | 295 |  |  | 144 | 148 | 192 | 196 | 262 | 262 | 127 | 127 | 209 | 213 | 191 | 199 |  |  |
| 16 | 149 | 149 | 196 | 196 | 323 | 323 | 137 | 137 | 131 | 131 | 248 | 260 | 150 | 158 | 283 | 291 | 274 | 274 | 144 | 156 | 188 | 192 | 266 | 266 | 123 | 123 | 213 | 213 | 191 | 195 | 210 | 210 |
| 17 | 141 | 149 | 196 | 196 | 319 | 335 | 137 | 137 | 131 | 131 | 260 | 260 | 150 | 150 | 275 | 295 | 290 | 294 | 144 | 160 | 180 | 184 | 270 | 274 | 127 | 127 | 213 | 217 | 191 | 191 | 190 | 214 |
| 18 | 149 | 153 | 196 | 196 | 319 | 323 | 133 | 137 | 131 | 147 | 256 | 260 | 150 | 158 | 275 | 299 | 270 | 294 | 144 | 144 | 180 | 184 | 266 | 274 | 127 | 127 | 217 | 221 | 191 | 191 | 214 | 218 |
| 19 | 141 | 145 | 196 | 196 | 323 | 335 | 133 | 137 | 131 | 147 | 260 | 296 | 150 | 158 | 283 | 295 | 290 | 298 | 144 | 152 | 180 | 180 | 270 | 274 | 127 | 127 | 217 | 221 | 191 | 203 | 190 | 218 |
| 20 | 141 | 153 | 196 | 196 | 319 | 323 | 133 | 137 | 127 | 131 | 260 | 296 | 150 | 158 | 295 | 299 | 290 | 294 | 144 | 160 | 180 | 184 | 266 | 274 | 127 | 127 | 217 | 221 | 191 | 191 | 190 | 210 |
| 21 | 141 | 145 | 188 | 196 | 311 | 323 | 133 | 133 | 127 | 147 | 256 | 260 | 154 | 158 | 283 | 287 | 270 | 294 | 144 | 160 | 180 | 184 | 266 | 266 | 127 | 127 | 213 | 221 | 191 | 191 | 186 | 218 |
| 22 | 145 | 149 | 188 | 192 | 311 | 319 | 137 | 137 | 147 | 147 | 252 | 252 | 150 | 154 | 283 | 291 | 298 | 302 | 144 | 160 | 188 | 192 | 266 | 274 | 127 | 127 | 205 | 213 | 191 | 195 | 190 | 210 |
| 23 | 145 | 149 | 188 | 192 | 323 | 327 | 133 | 133 | 147 | 147 | 248 | 252 | 150 | 154 | 283 | 291 | 286 | 298 | 144 | 160 | 188 | 192 | 266 | 274 | 127 | 127 | 209 | 221 | 191 | 195 | 222 | 222 |
| 24 | 141 | 145 | 188 | 196 | 311 | 335 | 133 | 133 | 131 | 147 | 260 | 260 | 154 | 154 | 275 | 287 | 270 | 270 | 140 | 160 | 184 | 188 | 266 | 266 | 123 | 127 | 213 | 213 | 191 | 203 | 186 | 190 |
| 25 | 141 | 149 | 196 | 196 | 323 | 335 | 133 | 133 | 131 | 135 | 256 | 276 | 154 | 158 | 295 | 299 | 282 | 286 | 144 | 156 | 184 | 192 | 250 | 266 | 135 | 139 | 209 | 209 | 191 | 195 | 214 | 218 |
| 26 | 145 | 149 | 188 | 196 | 327 | 335 | 133 | 137 | 139 | 139 | 252 | 256 | 142 | 142 | 287 | 287 | 274 | 278 | 140 | 164 | 188 | 188 | 266 | 266 | 127 | 139 | 213 | 213 | 191 | 203 | 214 | 214 |
| 27 | 145 | 149 | 188 | 196 | 315 | 331 |  |  | 131 | 139 | 252 | 252 | 142 | 142 | 279 | 279 | 278 | 282 | 144 | 144 | 184 | 188 | 254 | 262 | 123 | 123 | 209 | 209 | 203 | 203 | 214 | 218 |
| 28 | 145 | 149 | 188 | 196 | 315 | 331 | 133 | 137 | 131 | 139 | 256 | 276 | 142 | 154 | 279 | 287 | 282 | 282 | 152 | 160 | 184 | 188 | 254 | 262 | 127 | 139 | 209 | 209 | 195 | 199 | 214 | 214 |
| 29 | 149 | 149 | 196 | 196 | 319 | 335 | 133 | 137 | 131 | 139 | 252 | 272 | 142 | 142 | 275 | 287 | 282 | 282 | 148 | 148 | 184 | 188 | 262 | 270 | 123 | 139 | 209 | 209 | 195 | 199 | 194 | 214 |
| 30 | 145 | 145 | 188 | 188 | 315 | 331 | 133 | 137 | 147 | 147 | 252 | 256 | 154 | 158 | 275 | 287 | 278 | 282 | 140 | 148 | 192 | 192 | 262 | 266 | 123 | 123 | 209 | 209 | 191 | 199 | 214 | 214 |
| 31 | 145 | 145 | 196 | 196 | 315 | 331 | 133 | 133 | 131 | 155 | 252 | 272 | 142 | 142 | 279 | 279 | 282 | 286 | 144 | 160 | 184 | 192 | 262 | 262 | 123 | 139 | 209 | 213 | 199 | 203 | 214 | 214 |
| 32 | 145 | 145 | 196 | 196 | 319 | 327 | 133 | 137 | 139 | 155 | 252 | 264 | 154 | 158 | 279 | 279 | 282 | 282 | 152 | 152 | 184 | 184 | 254 | 262 | 127 | 131 | 209 | 209 | 191 | 191 | 214 | 214 |
| 33 | 145 | 145 | 188 | 188 | 315 | 319 | 133 | 137 | 131 | 139 | 252 | 276 | 154 | 154 | 287 | 287 | 278 | 282 | 144 | 164 | 184 | 184 | 262 | 266 | 127 | 139 | 209 | 213 | 191 | 191 | 214 | 214 |
| 34 | 145 | 149 | 188 | 192 | 315 | 331 | 133 | 133 | 131 | 131 | 252 | 276 | 142 | 142 | 275 | 275 | 282 | 282 | 148 | 148 | 184 | 188 | 254 | 270 | 123 | 127 | 209 | 213 | 191 | 199 | 190 | 214 |
| 35 | 145 | 145 | 200 | 200 | 315 | 335 | 137 | 137 | 131 | 139 | 264 | 264 | 142 | 142 | 275 | 279 | 282 | 282 | 148 | 152 | 184 | 184 | 262 | 266 | 127 | 131 | 209 | 209 | 191 | 199 | 214 | 214 |
| 36 | 145 | 145 | 188 | 192 | 315 | 331 | 133 | 137 | 131 | 155 | 260 | 276 | 142 | 154 | 279 | 287 | 286 | 286 | 152 | 152 | 184 | 184 | 262 | 270 | 123 | 127 | 209 | 217 | 191 | 191 | 190 | 214 |
| 37 | 145 | 145 | 192 | 196 | 315 | 323 | 133 | 133 | 139 | 139 | 252 | 264 | 142 | 158 | 275 | 279 | 282 | 282 | 140 | 160 | 184 | 188 | 266 | 266 | 123 | 123 | 209 | 213 | 191 | 199 | 190 | 214 |
| 38 | 145 | 145 | 188 | 192 | 315 | 331 | 129 | 133 | 131 | 139 | 252 | 272 | 142 | 158 | 279 | 279 | 282 | 282 | 144 | 152 | 184 | 184 | 262 | 266 | 123 | 131 | 213 | 213 | 191 | 203 | 190 | 214 |
| 39 | 145 | 149 | 188 | 192 | 319 | 331 | 133 | 133 | 139 | 155 | 252 | 252 | 142 | 154 | 275 | 279 | 282 | 286 | 148 | 160 | 184 | 188 | 266 | 270 | 123 | 139 | 209 | 213 | 199 | 199 | 214 | 214 |
| 40 | 145 | 145 | 188 | 188 | 319 | 319 | 133 | 137 | 131 | 131 | 252 | 264 | 142 | 142 | 279 | 287 | 282 | 282 | 148 | 156 | 192 | 192 | 266 | 266 | 123 | 127 | 209 | 217 | 199 | 199 | 214 | 214 |
| 41 | 145 | 145 | 188 | 188 | 319 | 331 | 133 | 137 | 131 | 131 | 252 | 264 | 142 | 142 | 279 | 287 | 282 | 282 | 152 | 160 | 188 | 192 | 266 | 266 | 123 | 131 | 209 | 217 | 199 | 199 | 214 | 214 |
| 42 | 145 | 149 | 188 | 200 | 315 | 335 | 133 | 133 | 131 | 131 | 252 | 276 | 142 | 154 | 275 | 279 | 278 | 282 | 148 | 160 | 184 | 192 | 262 | 270 | 123 | 139 | 209 | 213 | 199 | 199 | 214 | 214 |
| 43 | 141 | 145 | 196 | 196 | 315 | 315 | 129 | 137 | 131 | 135 | 252 | 260 | 154 | 158 | 275 | 291 | 282 | 290 | 148 | 156 | 180 | 184 | 262 | 266 | 127 | 139 | 205 | 205 | 187 | 195 | 210 | 218 |
| 44 | 145 | 149 | 200 | 200 | 315 | 319 | 133 | 133 | 131 | 151 | 252 | 268 | 146 | 146 | 287 | 303 | 290 | 290 | 152 | 160 | 168 | 168 | 266 | 270 | 127 | 127 | 209 | 209 | 191 | 199 | 218 | 218 |
| 45 | 149 | 149 | 196 | 196 | 315 | 331 | 133 | 137 | 131 | 131 | 252 | 268 | 146 | 150 | 275 | 295 | 290 | 290 | 152 | 156 | 168 | 180 | 270 | 270 | 131 | 135 | 209 | 221 | 187 | 195 | 194 | 214 |
| 46 | 149 | 149 | 196 | 196 | 323 | 327 | 133 | 133 | 131 | 151 | 252 | 268 |  |  |  |  |  |  | 160 | 160 | 184 | 184 | 258 | 262 | 127 | 135 | 209 | 209 | 199 | 199 | 194 | 218 |
| 47 | 149 | 149 | 196 | 196 | 327 | 327 | 133 | 137 | 131 | 135 | 252 | 256 | 146 | 158 | 303 | 307 | 290 | 290 | 152 | 156 | 184 | 188 | 262 | 262 | 127 | 135 | 209 | 209 | 199 | 199 | 202 | 218 |
| 48 | 149 | 149 | 196 | 196 | 319 | 323 | 137 | 137 | 131 | 135 | 268 | 276 | 154 | 158 | 283 | 299 | 290 | 294 | 144 | 148 | 184 | 184 | 262 | 266 | 135 | 135 | 209 | 209 | 195 | 195 | 186 | 214 |
| 49 | 149 | 149 | 196 | 196 | 315 | 315 | 129 | 133 | 131 | 131 | 248 | 264 | 154 | 154 | 271 | 291 | 290 | 290 | 148 | 156 | 184 | 192 | 266 | 274 | 135 | 135 | 209 | 213 | 199 | 199 | 186 | 194 |
| 50 | 145 | 149 | 196 | 196 | 323 | 331 | 129 | 133 | 131 | 131 | 260 | 268 | 134 | 146 | 291 | 291 | 290 | 294 | 152 | 160 | 184 | 188 | 266 | 270 | 135 | 135 | 209 | 217 | 199 | 203 | 210 | 214 |
| 51 | 145 | 149 | 196 | 200 | 315 | 327 | 129 | 137 | 131 | 135 | 252 | 252 | 150 | 154 | 275 | 307 | 270 | 298 | 148 | 152 | 168 | 180 | 270 | 270 | 127 | 135 | 201 | 209 | 191 | 203 | 186 | 194 |
| 52 | 137 | 149 | 196 | 196 | 323 | 323 | 133 | 133 | 131 | 135 | 248 | 268 | 154 | 154 | 291 | 299 | 286 | 290 | 144 | 152 | 192 | 192 | 266 | 266 | 135 | 135 | 209 | 209 | 195 | 203 | 186 | 218 |
| 53 | 149 | 149 | 196 | 196 | 323 | 327 | 133 | 133 | 131 | 151 | 252 | 268 | 154 | 158 | 271 | 279 | 278 | 286 | 160 | 160 | 184 | 184 | 258 | 262 | 127 | 135 | 209 | 209 | 199 | 199 | 194 | 218 |
| 54 | 145 | 149 | 196 | 200 | 319 | 323 | 133 | 133 | 131 | 135 | 248 | 252 | 154 | 158 | 283 | 299 | 290 | 290 | 148 | 160 | 184 | 192 | 266 | 266 | 119 | 135 | 209 | 213 | 187 | 199 | 186 | 194 |
| 55 | 145 | 145 | 192 | 196 | 315 | 327 | 137 | 141 | 139 | 139 | 260 | 268 | 146 | 146 | 279 | 283 | 282 | 286 | 148 | 152 | 188 | 188 | 262 | 270 | 135 | 135 | 209 | 213 | 199 | 203 | 218 | 218 |
| 56 | 149 | 149 | 196 | 196 | 319 | 319 | 137 | 137 | 131 | 151 | 252 | 276 | 154 | 158 | 283 | 291 | 282 | 286 | 152 | 152 | 192 | 196 | 262 | 270 | 135 | 135 | 209 | 209 | 203 | 203 | 190 | 218 |
| 57 | 145 | 145 | 196 | 196 | 315 | 331 | 133 | 133 | 131 | 155 | 252 | 272 | 142 | 142 | 279 | 279 | 282 | 286 | 144 | 144 | 184 | 192 | 262 | 262 | 123 | 139 | 209 | 213 | 199 | 203 | 214 | 214 |
| 58 | 145 | 145 | 196 | 200 | 315 | 327 | 129 | 133 | 131 | 135 | 252 | 252 | 158 | 158 | 303 | 307 | 286 | 286 | 152 | 152 | 180 | 188 | 258 | 270 | 119 | 131 | 209 | 209 | 199 | 199 | 210 | 218 |
| 59 | 145 | 145 | 196 | 196 | 323 | 323 | 129 | 133 | 131 | 131 | 256 | 296 | 138 | 158 | 299 | 307 | 274 | 298 | 148 | 148 | 184 | 188 | 258 | 270 | 131 | 135 | 209 | 213 | 191 | 195 | 210 | 222 |
| 60 | 141 | 149 | 192 | 196 | 319 | 323 | 133 | 137 | 131 | 139 | 248 | 268 | 154 | 154 | 283 | 299 | 282 | 290 | 148 | 152 | 184 | 192 | 262 | 266 | 119 | 135 | 209 | 217 | 195 | 199 | 186 | 194 |
| 61 | 137 | 145 | 196 | 196 | 323 | 323 | 129 | 133 | 131 | 135 | 248 | 268 | 154 | 158 | 295 | 299 | 270 | 286 | 152 | 152 | 184 | 192 | 266 | 270 | 131 | 135 | 209 | 221 | 195 | 199 | 190 | 210 |
| 62 | 145 | 145 | 196 | 196 | 319 | 323 | 129 | 141 | 135 | 139 | 260 | 268 | 158 | 158 | 275 | 295 | 270 | 282 | 148 | 152 | 180 | 184 | 262 | 270 | 119 | 131 | 209 | 217 | 191 | 199 | 186 | 190 |
| 63 | 145 | 149 | 196 | 196 | 331 | 331 | 129 | 129 | 143 | 151 | 260 | 264 | 158 | 158 | 279 | 279 | 278 | 282 | 144 | 160 | 188 | 192 | 266 | 278 | 139 | 139 | 205 | 209 | 191 | 199 | 190 | 206 |
| 64 | 149 | 149 | 196 | 200 | 319 | 319 | 129 | 129 | 131 | 131 | 276 | 276 | 134 | 146 | 291 | 307 | 286 | 286 | 144 | 152 | 184 | 184 | 262 | 270 | 135 | 135 | 209 | 209 | 195 | 203 | 210 | 218 |
