# Supplementary material for: Balancing selection and genetic drift at major histocompatibility complex class II genes in isolated populations of golden snub-nosed monkey (Rhinopithecus roxellana)
Source: BMC Evol Biol. 2012 Oct 19;12:207. doi: 10.1186/1471-2148-12-207 (PMC3532231; doi:10.1186/1471-2148-12-207)
Supplement: Additional file 2 — Table S2. MHC haplotype distributions in populations. [file 1471-2148-12-207-S2.doc]

**Table S2**

|  | SG | QL | SNJ | Number of individuals |
| --- | --- | --- | --- | --- |
| DQA1*01 | 10 | 10 | 12 | 31 |
| DQA1*02 | 5 | 6 | 7 | 18 |
| DQA1*03 | 10 | 4 | 1 | 15 |
| DQA1*04 | 1 | 2 | 7 | 10 |
| DQA1*05 | 0 | 5 | 0 | 5 |
| DQA1*06 | 5 | 3 | 0 | 8 |
| DQA1*07 | 3 | 0 | 0 | 3 |
| DQA1*08 | 0 | 3 | 0 | 3 |
| DQA1*09 | 0 | 1 | 0 | 1 |
| DQB1*01 | 15 | 8 | 4 | 26 |
| DQB1*02 | 11 | 7 | 0 | 18 |
| DQB1*03 | 1 | 4 | 7 | 12 |
| DQB1*04 | 8 | 7 | 0 | 15 |
| DQB1*05 | 0 | 0 | 9 | 9 |
| DQB1*06 | 5 | 0 | 0 | 5 |
| DQB1*07 | 0 | 1 | 4 | 5 |
| DQB1*08 | 0 | 1 | 0 | 1 |
| DQB1*09 | 0 | 2 | 0 | 2 |
| DQB1*10 | 0 | 1 | 0 | 1 |
| DQB1*11 | 0 | 1 | 0 | 1 |
| DQB1*12 | 0 | 2 | 0 | 2 |
| DQB1*13 | 1 | 0 | 0 | 1 |
| DQB1*14 | 0 | 1 | 0 | 1 |
| DQB1*15 | 1 | 0 | 0 | 1 |
| DQB1*16 | 0 | 0 | 1 | 1 |
