# Supplementary figures and images for: Balancing selection and genetic drift at major histocompatibility complex class II genes in isolated populations of golden snub-nosed monkey (Rhinopithecus roxellana)
Source: BMC Evol Biol. 2012 Oct 19;12:207. doi: 10.1186/1471-2148-12-207 (PMC3532231; doi:10.1186/1471-2148-12-207)

**Figure S1**


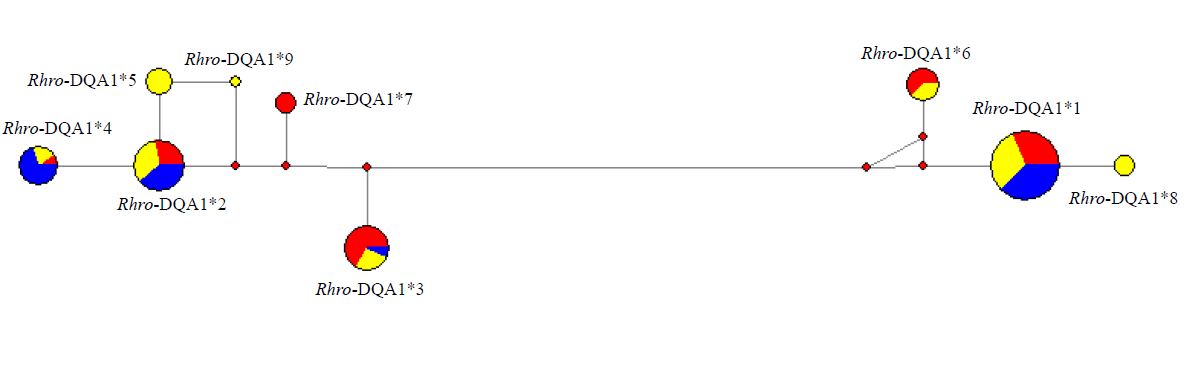


A


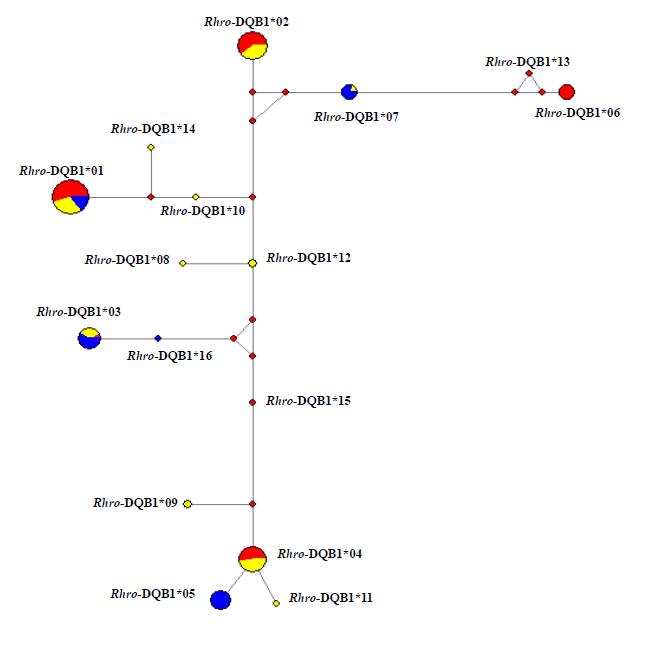


B

Supplement: Additional file 3 — Figure S1. Median-joining networks for MHC alleles of the snub-nosed monkeys. A) Network for DQA1 alleles; B) Network for DQB1 alleles. The circles represent alleles (SG population-red, QL population-yellow, SNJ population-blue), with the area proportional to the frequency of the alleles in the 3 populations. [file 1471-2148-12-207-S3.doc]

**Figure S2**


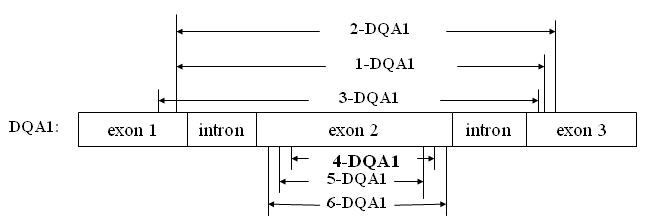

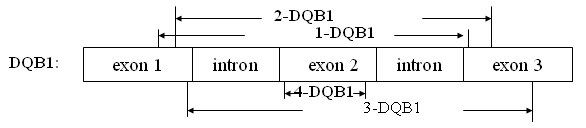

Supplement: Additional file 7 — Figure S2. Schematic representation of the position of cDNA primer sets used in the study of MHC II variation. [file 1471-2148-12-207-S7.doc]
