# Supplementary material for: Balancing selection and genetic drift at major histocompatibility complex class II genes in isolated populations of golden snub-nosed monkey (Rhinopithecus roxellana)
Source: BMC Evol Biol. 2012 Oct 19;12:207. doi: 10.1186/1471-2148-12-207 (PMC3532231; doi:10.1186/1471-2148-12-207)
Supplement: Additional file 4 — Table S3. Alignment of the deduced amino acid sequences of Rhro-DQA1 exon 2 sequences. Identical amino acids are shown by points, * represent ABS site, and sites revealed to be under significant selection in PAML are shown by dash. [file 1471-2148-12-207-S4.doc]

**Table S3**

| Haplotype | 1111111111222222222233333333334444444444555555555566666666667777777777888  1234567890123456789012345678901234567890123456789012345678901234567890123456789012  - - - - -- - - - - - - -- -- -  * * * * ** * * * * * * * ** * ** * |
| --- | --- |
| DQA1*01 | DHVASCGVNLYQTYGFSGQYTHEFDGDEQFYVDLGRKETAWRWPELSKFGGFDPQGALRNLATSKHNLNIMTKRYNSTAATN |
| DQA1*02 | .....Y......S..P............E.....E....V.QL.LF...RS...........VG.......I..S....... |
| DQA1*03 | .....Y...F..F..S..................E....V..L.LF.R.A..........I.VG.QT..M.I..S....... |
| DQA1*04 | .....Y......S..P............E.....E....V.QL.LF...RS...........VG......LI.CS....... |
| DQA1*05 | .....Y......S..P............E.....E....V.QL.LF...RS...........VG.......I..S...T... |
| DQA1*06 | ..............SL...F.............................................................. |
| DQA1*07 | .....Y.........L....S..................V..L.V..Q..S..A.F..T.I.VG.....FLI.SS....... |
| DQA1*08 | .................................................................................. |
| DQA1*09 | .....Y......S..P............E.....E....V.QL.LF....S...........VG.......I..S...T... |
