# Supplementary material for: Balancing selection and genetic drift at major histocompatibility complex class II genes in isolated populations of golden snub-nosed monkey (Rhinopithecus roxellana)
Source: BMC Evol Biol. 2012 Oct 19;12:207. doi: 10.1186/1471-2148-12-207 (PMC3532231; doi:10.1186/1471-2148-12-207)
Supplement: Additional file 5 — Table S4. Alignment of the deduced amino acid sequences of Rhro-DQB1 exon 2 sequences. Identical amino acids are shown by points, * represent ABS site, and sites revealed to be under significant selection in PAML are shown by dash. [file 1471-2148-12-207-S5.doc]

**Table S4**

| Haplotype | 1111111111222222222233333333334444444444555555555566666666667777777777888888  1234567890123456789012345678901234567890123456789012345678901234567890123456789012345  - - - - -- - - - - -  * * * ** ** * * * * ** * * * * * * * * |
| --- | --- |
| DQB1*01 | VQFKGMCYFTNGTERVRLVTRYIYNREEYARFDSDVWEYRAVTPLGRSSAEYWNSQKDVLEGTRAELDTVCRHNYQLELRTTLQR |
| DQB1*02 | .................G.A.........V......G..............H.........S....V.R..I............. |
| DQB1*03 | F........I......HH.N........FVG....LGV......M..PD.........I..S...VV.........S........ |
| DQB1*04 | Y....L...........S...........V......GV......Q..PD.........I..R.........M...EVAY.GI... |
| DQB1*05 | Y....L...........S...........V......GV......Q..PA.........I..R.........M...EVAY.GI... |
| DQB1*06 | Y.............H....A.HV.............GV........Q..........E...RK...................... |
| DQB1*07 | Y....L...........S...H.......V......G..............H.........S....V.........S........ |
| DQB1*08 | .....L......................NV......G..........PD............S....V.........S........ |
| DQB1*09 | Y....L...............H......F.......G.......Q..PD.........I..R.........I...EVAY.GI... |
| DQB1*10 | .....L.......................V......G........................S....V.........S........ |
| DQB1*11 | Y....L...........S...........V......GV......Q..PD.........I..R..E......M...EVAY.GI... |
| DQB1*12 | .....L.......................V......G..........PD............S....V.........S........ |
| DQB1*13 | Y.............H....A.HV...D.........GV........Q..........E...RK...................... |
| DQB1*14 | .....L.............A.........V......G........................S....................... |
| DQB1*15 | .....L...........S...........V......GV......Q..PD.........I..R.........M............. |
| DQB1*16 | F...............HH.N........FVG....LGV......M..PD.........I..S...VV.........S........ |
