# Supplementary material for: Balancing selection and genetic drift at major histocompatibility complex class II genes in isolated populations of golden snub-nosed monkey (Rhinopithecus roxellana)
Source: BMC Evol Biol. 2012 Oct 19;12:207. doi: 10.1186/1471-2148-12-207 (PMC3532231; doi:10.1186/1471-2148-12-207)
Supplement: Additional file 6 — Table S5. cDNA primer sets for DQA and DQB (primer sets with * were those that amplified successfully in cDNA). Their location within the gene is shown in Additional file 7: Figure S2. [file 1471-2148-12-207-S6.doc]

**Table S5**

| Primer set | Sequence(5'-3') |
| --- | --- |
| 1-DQA1F | ATGATCCTAAACAAAGCTCTG |
| 1-DQA1R | GAAAACACTGTGACCTCAGGAACCT |
| 2-DQA1F | ATGATCCTAAACAAAGCTCTG |
| 2-DQA1R | CCACAAGACAGATGAGGGTGTTGGG |
| 3-DQA1F | CAGTATGATCCTAAACAAAGCTC |
| 3-DQA1R | TGAGGTAACTGATCTTGAAGAAGGA |
| *4-DQA1F | GGTGTAAACTTGTACCAG |
| *4-DQA1R | TGGTAGCAGCGGTAGAGTT |
| 5-DQA1F | CCTCTTGCGGTGTAAACTTGTACCAG |
| 5-DQA1R | GCTCCAACTCTACCGCTGCTACCAA |
| 6-DQA1 | CTGACCACGTTGCCTCTTGCGGTGTAA |
| 6-DQA1 | AACTCTACCGCTGCTACCAA |
| *1-DQB1F | ATGTCTTGGAAGAAGGCTTTGCGGA |
| *1-DQB1R | GGATGGGGAGATGGTCACTGTGGGC |
| *2-DQB1F | CCTTGATGCTGGCGATGCTGAGCAC |
| *2-DQB1R | CCGGACTTTGATCTGGCTTGGATAGAA |
| 3-DQB1F | GTGGCTGAGGGCAGAGACTCTCC |
| 3-DQB1R | GGGAGTCATTTCCAGCATCACCAGGAT |
| *4-DQB1F | CCAGTTTAAGGGYATGTGCTACTT |
| *4-DQB1R | CAGGACKTCCTTCTGGCTGTTCCAGT |
